# Horizon Scanning in Tissue Engineering Using Citation Network Analysis

**DOI:** 10.1007/s43441-023-00529-x

**Published:** 2023-05-18

**Authors:** Kouhei Otsuka, Takuya Takata, Hajime Sasaki, Mayumi Shikano

**Affiliations:** 1 Faculty of Pharmaceutical Sciences, Tokyo University of Science, Tokyo, Japan; 2 Institute for Future Initiatives, The University of Tokyo, Tokyo, Japan

**Keywords:** Horizon scanning, 3D bio-printing, Tissue engineering, Citation network analysis, Bibliometric, Regulatory science, Text mining, Scaffold

## Abstract

**Background:**

Establishing a horizon scanning method is critical for identifying technologies that require new guidelines or regulations. We studied the application of bibliographic citation network analysis to horizon scanning.

**Objective:**

The possibility of applying the proposed method to interdisciplinary fields was investigated with the emphasis on tissue engineering and its example, three-dimensional bio-printing.

**Methodology and Results:**

In all, 233,968 articles on tissue engineering, regenerative medicine, biofabrication, and additive manufacturing published between January 1, 1900 and November 3, 2021 were obtained from the Web of Science Core Collection. The citation network of the articles was analyzed for confirmation that the evolution of 3D bio-printing is reflected by tracking the key articles in the field. However, the results revealed that the major articles on the clinical application of 3D bio-printed products are located in clusters other than that of 3D bio-printers. We investigated the research trends in this field by analyzing the articles published between 2019 and 2021 and detected various basic technologies constituting tissue engineering, including microfluidics and scaffolds such as electrospinning and conductive polymers. The results suggested that the research trend of technologies required for product development and future clinical applications of the product are sometimes detected independently by bibliographic citation network analysis, particularly for interdisciplinary fields.

**Conclusion:**

This method can be applied to the horizon scanning of an interdisciplinary field. However, identifying basic technologies of the targeted field and following the progress of research and the integration process of each component of technology are critical.

**Supplementary Information:**

The online version contains supplementary material available at 10.1007/s43441-023-00529-x.

## Introduction

The development of medical products in which the latest innovative technologies are applied has attracted considerable attention. However, applying conventional development/evaluation concepts and regulatory frameworks to these products is sometimes not considered feasible. Therefore, developing new guidelines/regulations in advance is critical for the efficient development of products as well as for quick patient access and improved benefit/risk ratios of the products. Thus, innovative technologies that can be applied to pharmaceuticals and medical devices should be identified at early stages. Activities conducted for the early identification of such innovative technologies is called horizon scanning [1–3].

According to the International Coalition of Medicines Regulatory Authorities (ICMRA) on the strategic priority in innovation, horizon scanning refers to broad-reaching information-gathering and monitoring activities conducted for anticipating emerging products and technologies and potentially disruptive research avenues [4]. The importance of sharing the implementation status and establishing methodologies in each country is mentioned [4]. However, Hines reported that most horizon scanning examples in medicinal fields have been conducted in a nonautomated manner that relied on expert opinion and lack objectivity [5]. Furthermore, nonautomated methods cannot respond to technological innovations that increase in complexity and volume of information [5]. Therefore, a horizon scanning methodology that systematically utilizes large-scale information should be devised.

Bibliometric analysis is a method in which bibliographic information, such as academic articles are analyzed metrologically. Bibliometric analyses have attracted considerable research attention [6–11]. However, most studies have focused on the history of research developments and/or current research progress in targeted topics. Limited studies have focused on gaining insight into future trends. Therefore, we focused on applying bibliometric analyses to horizon scanning, which can be used for obtaining extensive literary information on science, technology, and research systematically.

When using bibliographic information metrologically, considering the differences in development history and tendency of publication and citation according to each research field is crucial. In previous studies on citation network analysis in the fields of immunology and artificial intelligence (AI)-equipped medical devices, we suggested that the method is useful as a primary screening tool for extracting research topics for horizon scanning. Furthermore, we recommended that the results of this method should be interpreted according to the development history of each research field. Research activities in the field of immunology have focused on various reactions and molecules for an extended period, whereas the research and development of artificial intelligence (AI)-equipped medical devices have progressed rapidly [12–15]. The Leiden Manifesto issued by the Centre for Science and Technology Studies at Leiden University in 2015 also indicated that bibliometric analysis should be performed with due consideration for differences between research fields [16].

However, most studies aimed at using bibliometric analyses for horizon scanning have focused on setting models and indicators of the analytical procedure, whereas limited studies have focused on the differences in developmental history, publication trends, and citation trends in each research field [17–21].

We anticipated that the results of the citation network analysis for interdisciplinary research activities could have distinct characteristics compared with other research fields. Therefore, in this study, we focused on tissue engineering, an interdisciplinary research activity that includes cell physiology, cell biochemistry, biophysics, and materials engineering, with the emphasis on three-dimensional (3D) bio-printing as a specific case in tissue engineering. Notably, 3D bio-printing is a technology that focuses on creating 3D tissues/organs by printing cells and hydrogels with a 3D printer [22, 23]. This technology is characterized by complex manufacturing processes and source materials and has both pharmaceutical and medical device aspects. This technology has been the subject of a case study in ICMRA and is a critical topic of research [2].

In this study, we first conducted a retrospective analysis of 3D bio-printing to confirm whether the results of citation network analysis reflect the development history of tissue engineering. Next, we extracted clusters containing recently published papers to explore latest research topics. Subsequently, we discussed the aspects to consider while performing citation network analysis for the horizon scanning of interdisciplinary fields.

## Methods

### Extraction of Paper Data for Analysis

Because the research activity of 3D bio-printing generally consists of three research fields; tissue engineering and regenerative medicine, biofabrication and additive manufacturing [22–24], queries to acquire articles for citation network analysis were set as follows: “((tissue OR organ) AND engineering) OR (regenerative AND (medicine OR therapy)) OR ((bio* AND fabrication) OR biofabrication) OR ((rapid AND prototyping) OR (rapid AND manufacturing) OR (additive AND fabrication) OR (additive AND manufacturing)).” By searching for fields in a “Topic” in this query, we obtained 233,968 papers published between January 1, 1900 and November 3, 2021 (1900–2021) from the Science Citation Index (SCI) Expanded, Social Sciences Citation Index (SSCI), and Emerging Sources Citation Index (ESCI) by Web of Science Core Collection (Web of Science, Clarivate Analytics).

### Citation Network Analysis

**Figure 1:**
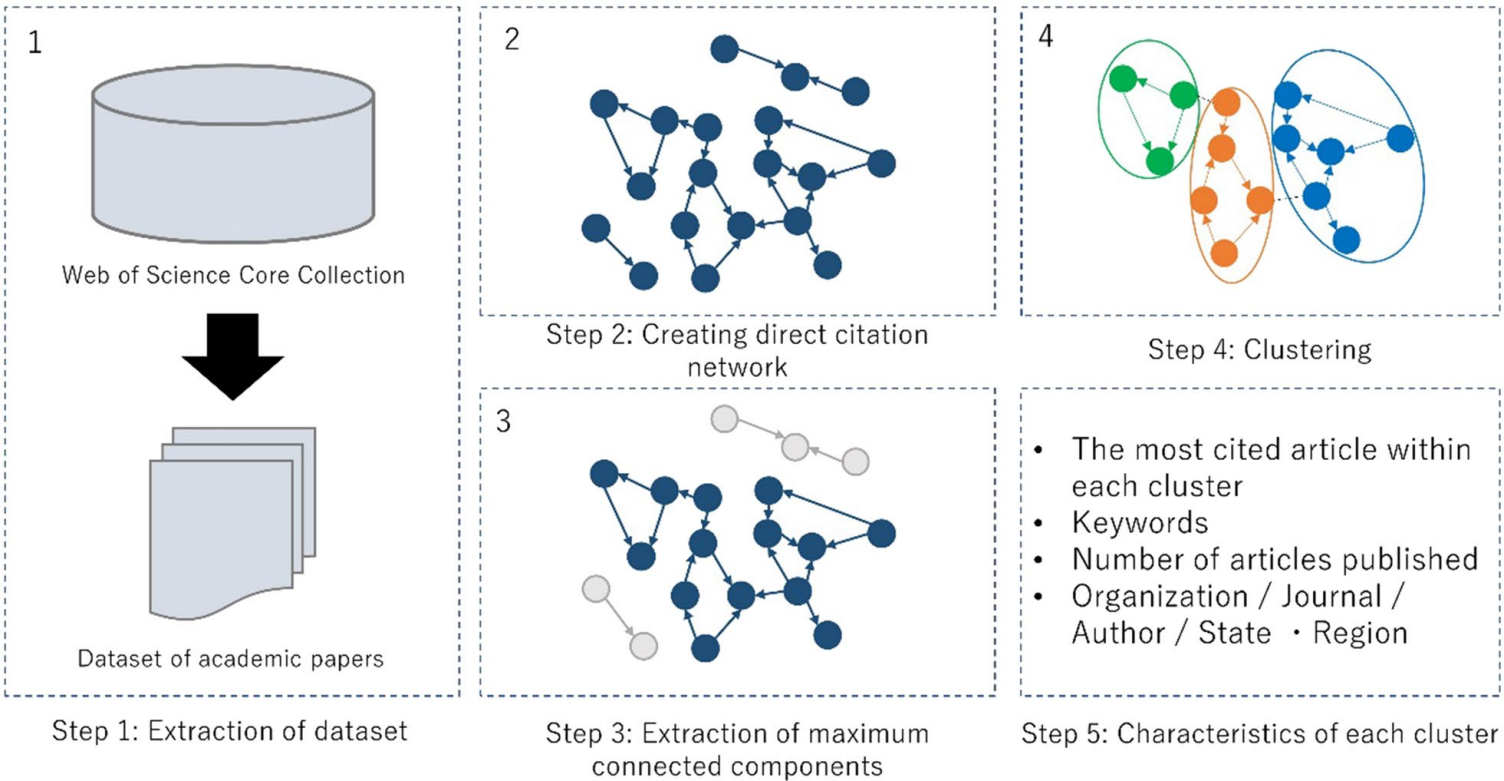
Procedure for Conducting Citation Network Analysis [25, 26]. Step 1: obtain article data including the citation information from the Web of Science Core Collection. Step 2: the article data including citation information is considered to be a network structure with articles and direct citation relationships as nodes and links. Step 3: extract consecutive networks, including the largest nodes from the formed network structure. Step 4: perform clustering using the Louvain method. Step 5: Obtain information by methods such as text mining on the clusters formed.

We performed citation network analysis using the following procedure as displayed in Fig. 1: the large dataset of articles was considered as a network structure [25, 26]. Here, articles and citation relationships were nodes and links, respectively. First, the largest consecutive citation network with the largest number of nodes was extracted from this network structure. Nodes and links were excluded. Subsequently, the extracted network structure was analyzed using the Louvain method to maximize the modularity (Q value) of links. The nodes were categorized into clusters, such that many links existed within each cluster and fewer links existed between clusters [27–29]. The *Q* value that represents modularity is expressed as follows:

$$Q=\sum_{i}\left({e}_{ii}-{a}_{i}^{2}\right),$$

where ${e}_{ii}$ is the ratio of links that exist inside cluster $i$ to the entire network and ${a}_{i}^{2}$ is the expected value of the ratio of links existing inside cluster $i$, which is calculated from the number of links in the entire network structure. The formed clusters were numbered from No. 1 in the descending order of the number of contained nodes.

The contents of clusters were confirmed from keywords pertaining to the characteristic of the clusters and articles that are typically cited within each cluster. Furthermore, the term frequency inverse cluster frequency ($TFICF$) was used to determine the characteristic of each cluster. The $TFICF$ for the word $j$ in cluster $i$ is expressed as follows:

$$\text{TFICF}={tf}_{i,j}\bullet {icf}_{i}={tf}_{i,j}\cdot \text{log}(N/{cf}_{i}),$$

where $t{f}_{i, j}$ is the appearance ratio of the word $j$ to all words in cluster $i$,$N$ is the total number of clusters, and $c{f}_{j}$ is the number of clusters containing the word $j$. Therefore, keywords with large TFICF frequently appear in specific clusters, which indicates that the keywords based on the characteristic of each cluster can be confirmed. This index is calculated for each cluster by applying term frequency inverse document frequency (TFIDF), which is an index used for each document. This method has been used in past analyses [12–15, 30].

The topic of the cluster was assessed by reviewing most cited articles within each cluster.

### Tracking the Time Series of Key Articles

To evaluate whether the clustering results reflect the research history of 3D bio-printing, we tracked milestone articles in the development of 3D bio-printers. Table 1 details the list of tracked articles. Articles A to D are milestone publications in the development of 3D bio-printing technologies; articles A [31] and B [32] were cited in review articles that mentioned the development history of 3D bio-printing, whereas articles C [33] and D [34] were obtained by searching in Web of Science Core Collection for “organ AND printing” or “bioprinting” and were not review articles with a high number of citations. Articles E to N discuss activities relating to 3D bio-printed specific tissue products aiming at clinical application [35–44].

**Table 1:** Key Articles Tracked in Time Series Analysis.

Label	Title	Year of Publication
A	Biofabrication: a twenty-first century manufacturing paradigm [31]	2009
B	Organ printing: computer-aided jet-based 3D tissue engineering [32]	2003
C	Organ printing: tissue spheroids as building blocks [33]	2009
D	Scaffold-free vascular tissue engineering using bioprinting [34]	2009
E	In vitro biofabrication of tissues and organs [35]	2013
F	EphB signaling directs peripheral nerve regeneration through Sox2-dependent Schwann cell sorting [36]	2010
G	Development of an immunodeficient pig model allowing long-term accommodation of artificial human vascular tubes [37]	2019
H	Mechanism of peripheral nerve regeneration using a bio 3D conduit derived from normal human dermal fibroblasts [38]	2021
I	Toward engineering functional organ modules by additive manufacturing [39]	2012
J	Principles of the Kenzan method for robotic cell spheroid-based three-dimensional bioprinting [40]	2017
K	The efficacy of a scaffold-free bio 3D conduit developed from autologous dermal fibroblasts on peripheral nerve regeneration in a canine ulnar nerve injury model: a preclinical proof-of-concept study [41]	2019
L	Simultaneous regeneration of full-thickness cartilage and subchondral bone defects in vivo using a three-dimensional scaffold-free autologous construct derived from high-density bone marrow-derived mesenchymal stem cells [42]	2014
M	Scaffold-free tubular tissues created by a bio-3D printer undergo remodeling and endothelialization when implanted in rat aortae [43]	2015
N	The efficacy of a scaffold-free Bio 3D conduit developed from human fibroblasts on peripheral nerve regeneration in a rat sciatic nerve model [44]	2017

Articles A–D were milestones of the development on 3D bio-printing technologies, whereas Articles E–N were related to 3D bio-printed products for clinical applications

## Results

### Result of Citation Network Analysis

As a result of the citation network analysis of 233,968 articles obtained from the Web of Science Core Collection, 197,948 (84%) formed 78 clusters. The top ten clusters contained more than 80% of all articles or contained key articles. Table 2 details the topic of each cluster as well as the title of hub-paper articles most cited, and high TFICF keywords characteristic to the cluster. Clusters related to the scaffold for tissue engineering (cluster 1), stem cells (cluster 2), and 3D bio-printing technologies (cluster 3) were observed.

**Table 2:** Information on the Top Ten Clusters with the Number of Articles Included.

Cluster No.	Average Year	Number of Publications	Major Topic	Keywords	Title of the Hub-Paper
Cluster 1	2014.5	29,186	Scaffold for tissue engineering [45, 46]	Scaffold, bone, tissue engineering, nanofibers, composite, hydroxyapatite, chitosan, porous, collagen, pcl	Tissue engineering [45]
Cluster 2	2014.1	25,067	Stem cells [47, 48]	Stem cell, bone, mesenchymal stem cell, differentiation, tissue engineering, scaffold, marrow, therapy, stromal, adipose	Multilineage cells from human adipose tissue: Implications for cell-based therapies [47]
Cluster 3	2015.5	19,199	3D bio-printing [22, 49]	Hydrogel, scaffold, tissue engineering, stem cell, culture, bioprinting, nerve, alginate, delivery, printing	3D bioprinting of tissues and organs [22]
Cluster 4	2016.2	18,203	Additive manufacturing [50, 51]	Printing, additive manufacturing, composite, mechanical, prototyping, polymer, ceramic, fused, fabrication, material	Additive manufacturing (3D printing): A review of materials, methods, applications and challenges [50]
Cluster 5	2015.5	17,762	Conductive materials [52, 53]	Electrode, electrochemical, graphene, nanoparticles, biosensor, detection, carbon, glucose, film, fabrication	Conductive polymers: Towards a smart biomaterial for tissue engineering [52]
Cluster 6	2014.3	15,865	Microfluidics [54, 55]	Microfluidic, surface, fabrication, device, sensor, film, chip, substrate, array, detection	Rapid prototyping of microfluidic systems in poly(dimethylsiloxane) [54]
Cluster 7	2018	14,243	Metal additive manufacturing [56, 57]	Laser, additive manufacturing, alloy, powder, microstructure, selective laser melting, mechanical property, metal, titanium	Additive manufacturing of metallic components—Process, structure and properties [56]
Cluster 8	2013.5	11,200	Vascular and cardiac patches and decellularized tissue [58, 59]	Scaffold, tissue engineering, graft, valve, vascular, stem cell, decellularized, collagen, matrix, endothelial	An overview of tissue and whole organ decellularization processes [58]
Cluster 9	2014.7	8137	Tissue engineering for muscle tissue [60, 61]	Cardiac, stem cell, muscle, tissue engineering, heart, cardiomyocytes, myocardial, scaffold, skeletal, cell sheet	Engineered heart tissue grafts improve systolic and diastolic function in infarcted rat hearts [60]
Cluster 10	2012.9	7816	Tissue engineering for cartilage [62, 63]	Scaffold, chondrocytes, tissue engineering, collagen, articular cartilage, stem cell, repair, bone, culture, mesenchymal stem cell	Articular cartilage repair: basic science and clinical progress. A review of the current status and prospects [62]

The average publication year of the articles included in each cluster (average year), number of articles included in each cluster (number of articles), the topic of each cluster (major topic), keywords with TFICF in each cluster (top keywords), and the most cited articles within each cluster (hub-paper) are presented in the table

### Tracking the Time Series of Key Articles

**Figure 2:**
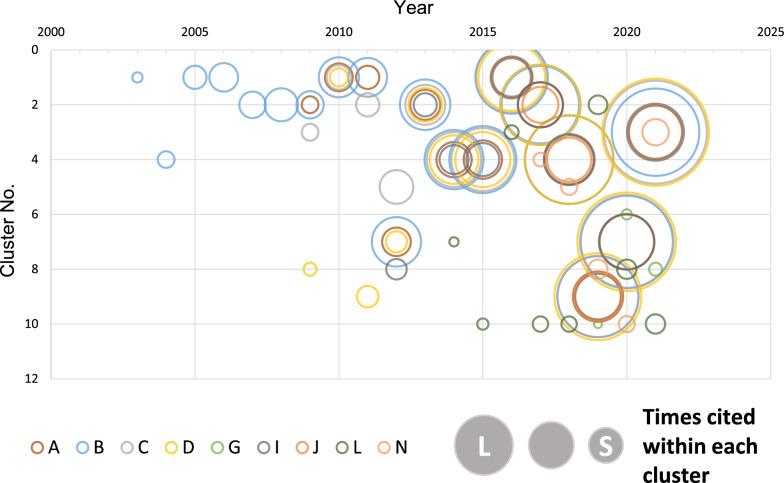
Tracking the time series of key articles. The articles published by each year are obtained from the database and an analysis is conducted with time. The horizontal axis represents the year of publication of the articles in the dataset and the vertical axis represents the cluster number. As displayed in the figure, the cluster contains the main articles over time. The size of the circle indicates the number of citations in each cluster containing the key articles.

We tracked key articles presented in Table 1, from 2000 to 2021, as displayed in Fig. 2.

Articles A to D [31–34], which are the milestones in the development of 3D bio-printing technologies, were each located in different clusters until 2012, and the number of times cited within each cluster increased gradually. Since 2013, articles detected in the same cluster and times cited within the cluster have risen sharply, which indicate that the attention to each article increased rapidly.

Regarding the articles that focused on the clinical applications of 3D bio-printed products, Article I [39], which is a review of 3D bio-printing, and Article J [40], which is related to technologies for preparation of 3D bio-printed products, are always in the same cluster with Articles A to D since 2013. Article N [44] on the transplantation of neural 3D bio-printed conduits into rats was in a different cluster from that of Articles A to D except in 2021. Article G [37] on transplantation of 3D bio-printed blood vessels into pigs and Article L [42] on preparation cartilage tissue by spheroid placement and aggregation were always detected in different clusters than Articles A to D. Articles F, H, K, and M were not included in 233,968 articles acquired in Method 2.1, but they were included in the Web of Science Core Collection. Article E was not included in the Web of Science Core Collection.

### Recent Research Trends in Tissue Engineering

**Figure 3:**
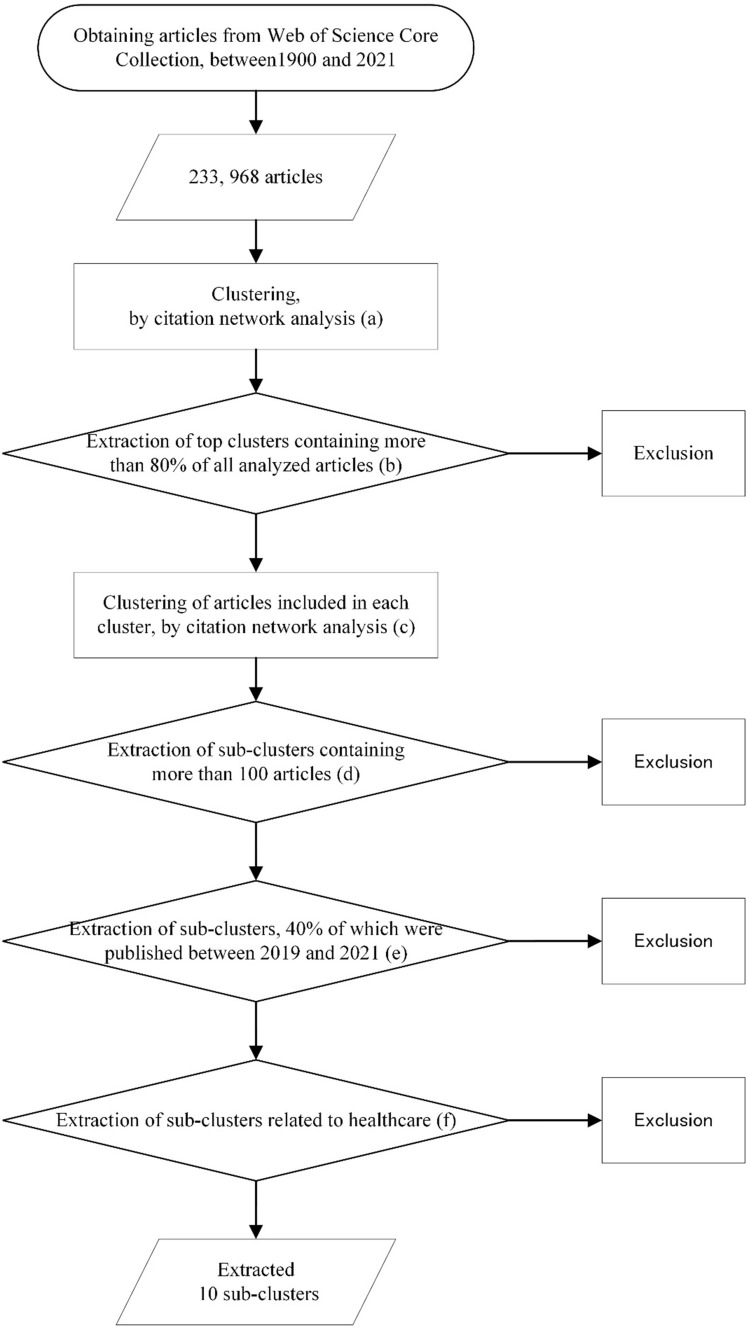
Procedure for investigating recent research trends in tissue engineering. (a) Obtained clusters by the citation network analysis of articles published by 2021, as summarized in Table 1. (b) Each cluster re-analyzed from cluster 1–9 that contain 80% of all articles analyzed. (c) Obtained sub-clusters. (d)–(e) Extracted sub-clusters containing more than 100 articles and over 40% of which were published between 2019 and 2021. (f) Further extracted sub-clusters that related to healthcare.

To investigate recent research progress, citation network analysis was conducted again for articles in clusters 1–9, which contained 80% or more articles of all articles analyzed, and extracted sub-clusters with a rapid increase in the number of articles between 2019 and 2021. We extracted the sub-clusters containing over 100 articles, over 40% of which were published between 2019 and 2021, and are related to healthcare based on *TFICF* and the content of the article with a high number of citations in each cluster. We focused on the trends during 3 years, from 2019 to 2021, considering that shorter periods does not allow stable extraction of sub-clusters, and 4 years or longer periods resulted in smaller differences between sub-clusters, where the increase in the number of articles has decreased in recent years. Furthermore, academic articles have been reported to be the most cited references for articles published in the last 3 years [64]. The percentage criterion of 40% was strictly set to specifically extract sub-clusters with rapidly increasing research activities (see Supplementary Material 1–3). These procedures on the extraction of research topics are displayed in Fig. 3.

The topics, hub-paper, and high $TFICF$ keywords of selected sub-clusters are indicated in Table 3. Sub-clusters related to scaffold for tissue engineering (sub-cluster 1-10, 1-12, 5-13, 7-1), hydrogel (sub-cluster 3-3), 3D bio-printing technologies (cluster 3-2), microfluidics (sub-cluster 4-1, 4-2), mesenchymal stem cell (sub-cluster 2-2), and tissue engineering for heart (sub-cluster 9-3) are observed.

**Table 3:** Sub-clusters with High Research Activity.

Sub-Custer No.	Number of Publications	Rate of Publications Since 2019	Topic	Keywords	Title of the Hub-Paper
Sub-cluster 1-10	340	0.444	Melt electrospinning for scaffold in tissue engineering [65, 66]	Scaffold, melt, printing, melt electrospinning writing, bone, electrohydrodynamic, fiber, electrospinning, melt electrospinning, jet	Direct writing by way of melt electrospinning [65]
Sub-cluster 1-12	178	0.466	Porous zein for scaffold in tissue engineering [67, 68]	Scaffold, zein, bone, tissue engineering, porous, mechanical, nanocomposite, nanoparticles, mechanical property, nanocomposite scaffold	Basic study of corn protein, zein, as a biomaterial in tissue engineering, surface morphology and biocompatibility [67]
Sub-cluster 2-2	3771	0.407	Mesenchymal stem cells suppress immune rejection [69, 70]	Msc, stem cell, mesenchymal, stem, cell, mesenchymal stem, mesenchymal stem cell, bone, stromal, stromal cell	Mesenchymal stem cells for treatment of steroid-resistant, severe, acute graft-versus-host disease: a phase ii study [69]
Sub-cluster 3-2	4214	0.439	Injectable hydrogel for tissue engineering [71, 72]	Hydrogel, tissue engineering, tissue, scaffold, delivery, injectable, polymer, gel, gelatin, drug delivery	Highly stretchable and tough hydrogels [71]
Sub-cluster 3-3	2776	0.478	3D bio-printing [22, 73]	Bioprinting, hydrogel, printing, scaffold, tissue, construct, bioink, cell, tissue engineering, bioinks	3D bioprinting of tissues and organs [22]
Sub-cluster 4-1	2680	0.508	3D-printing in microfluidics [74, 75]	Printing, printed, additive manufacturing, ink, manufacturing, device, electrode, additive, sensor, fabrication	The upcoming 3D-printing revolution in microfluidics [74]
Sub-cluster 4-3	2429	0.548	3D printing technology that considers time changes due to environmental stimuli [76, 77]	Printing, soft, actuator, robot, polymer, composite, additive manufacturing, shape, mechanical, material	Biomimetic 4D printing [76]
Sub-cluster 5-13	315	0.501	Conductive polymers for tissue engineering [78, 79]	Piezoelectric, pvdf (polyvinylidene fluoride), scaffold, fluoride, vinylidene, nanogenerator, tissue engineering, vinylidene fluoride, energy, bone	Piezoelectric polymers as biomaterials for tissue engineering applications [79]
Sub-cluster 7-1	2642	0.484	Porous metal for bone scaffold [80, 81]	Implant, porous, alloy, bone, scaffold, titanium, lattice, mechanical, laser, mechanical property	Topological design and additive manufacturing of porous metals for bone scaffolds and orthopaedic implants: a review [80]
Sub-cluster 9-3	1221	0.457	Cardiac tissue engineering using induced pluripotent stem cells [82, 83]	Cardiac, cardiomyocytes, heart, stem cell, stem, pluripotent, pluripotent stem, pluripotent stem cell, cell, tissue	Biowire: a platform for maturation of human pluripotent stem cell-derived cardiomyocytes [82]

Healthcare-related sub-clusters containing over 100 articles, and more than 40% of articles were published between 2019 and 2021. The topic of each sub-cluster was estimated based on TFICF and the content of the hub-paper. The number of articles (number of publication), the percentage of articles published in 2019–2021 (rate of publications since 2019), keywords with the high term frequency inverse cluster frequency in each cluster (top keywords), and the most cited articles in each cluster (hub-paper) is indicated in Table 3

## Discussion

In this study, the dataset of articles in the three research fields, namely tissue engineering and regenerative medicine, additive manufacturing and biofabrication [22–24] were acquired for analyzing 3D bio-printing from the Web of Science Core Data Collection. The citation network analysis of the obtained articles resulted in clusters pertaining to the technologies constituting this field, such as scaffolds used in tissue engineering, stem cells, and 3D bio-printing. A notable characteristic of the analysis result is that several clusters, including the largest cluster, are related to scaffolds used in tissue engineering, such as conductive materials and decellularized tissues. This phenomenon indicated that topics related to base materials that constitute the scaffold have received considerable attention in the research field of tissue engineering in addition to cell physiology and cell biochemistry, which require expertise distinct from that of scaffolds. This phenomenon is the major cause of obtaining articles for analysis from the Web of Science Core Data Collection; we found far less papers on scaffolds in PubMed (data not shown).

Next, we tracked the key articles for milestones in the development of 3D bio-printing and confirmed that the development history of 3D bio-printing is reflected in the results of the citation network analysis as displayed in Fig. 2.

Silvia et al. reported that the number of published patents on 3D bio-printing remained less than one per year until 2012; however, the number increased sharply since 2013, with 20 patents published annually in 2015, over 50 in 2018, and over 120 in 2020 [7]. They revealed that most patents were related to scaffolds and bio-printers in the early years. Ozbolat et al. indicated that the extrusion-based bio-printers, which is the most commonly used type in 3D bio-printing, has been available since 2012 [84]. Furthermore, Nakamura noted that the global boom in 3D printers began in 2012–2013 and since then 3D bio-printing attracted considerable research attention. Thus, the research and development of 3D bio-printing has developed rapidly with the progress of 3D printing technology since 2012–2013 [85]. The results in Fig. 2 were consistent with the rapid development centering on 3D printing technology mentioned above; Articles A to D [31–34], which are milestones in the development history of 3D bio-printing technologies, are located in the same cluster and times cited within each cluster as well as the number of articles in the cluster. Publication of such articles increased sharply since 2013, which suggests the rapid increase in research activity focusing on 3D bio-printing. In 2014 and 2019, the number of articles in the clusters, which contains key articles decreased considerably. However, the number of times the key article was cited did not decrease; the topic of the articles in the cluster suggests that an independent and specialized cluster for 3D bio-printing was formed.

Regarding 3D bio-printed product development for incorporation into clinical application, we tracked Articles E to N [35–44] related to preparation of organ or tissue including Article I; a review of 3D bio-printing [39], Article J on 3D printing technology [40], Article N on the transplantation of a nerve conduit created from 3D bio-printed spheroids into a rat [44], Article G on the transplantation of a blood vessel created from 3D bio-printed spheroids into pigs [37], and Article L on the creation of cartilage tissue by placing and aggregating spheroids [42].

Our results in Fig. 2 exhibited that Articles E to N were in a distinct cluster than Articles A to D [39, 40] except for Articles I and J on bio-printing technology in 2021.

We performed sub-clustering on clusters 3, 8, and 10 to confirm in which sub-clusters Article G, L, and N were detected and found that Article N [44] was in the sub-cluster on nerve regeneration, Article G [37] in the sub-cluster on rejection, and Article L [42] in the sub-cluster on cartilage tissue engineering (See Supplementary Material). This result could be attributed to the fact that Article N [44] cited many papers on regeneration of cartilage tissue, Article G [37] cited papers on the creation of an immunocompromised pig model, and Article L [42] cited papers on regeneration of cartilage tissue (data not shown). Thus, citation network analysis results are sometimes strongly influenced by the scope of the article and the interest of the authors, particularly in the interdisciplinary research fields. The clinical trials of conduit and blood vessels prepared by 3D bio-printers have been registered in the Japanese clinical trial registry, which shows that investigator-initiated clinical trials in the Saga University Hospital started in November 2019 for blood vessels and in the Kyoto University Hospital, it started in November 2020 for nerve conduits [86, 87]. In both products, spheroids were placed on a needle by the bio-printer, Regenova^®^, to create tubular tissue-like products; then, the tubular structure was strengthened by the perfusion culture to prepare a tissue for transplantation. Regarding neural conduits, cytokines released from fibroblasts used for spheroids preparation is one of the critical factors for inducing the regeneration of axon in rats [43, 44]. This result indicated that preparation of 3D bio-printed products for clinical application requires not only printing technology but also elaboration on spheroids production, which includes spheroids fusion conditions and factors for cell proliferation and differentiation.

These results confirmed that the field of tissue engineering, including 3D bio-printers, utilizes diverse technologies, including bio-printing and materials engineering related to cell scaffolds as well as cell physiology such as cell proliferation and differentiation and cell–cell interaction. Therefore, these methods have been integrated in clinical applications. Furthermore, articles on these technologies, which are the building blocks of this field, form separate clusters depending on their technological background, regardless of the application of those technologies to the product. Therefore, when targeting interdisciplinary fields such as tissue engineering, understanding all trends in product development toward clinical applications, such as the integration and application of individual basic technologies only by citation network analysis, is difficult.

This study revealed that the analysis of the interdisciplinary fields is characterized by the distribution of articles in clusters of their basic technologies, which differs from AI-based medical products and immunology fields [12, 13]. When applying horizon scanning based on the citation network analysis to an interdisciplinary research field confirming the development of the product or medical technology under consideration and evaluating not only the research trends in nonclinical studies toward clinical application but also trends in basic technologies that constitute the product or medical technology is necessary.

The analysis on the latest research trends in tissue engineering detected several technologies constituting the research field rather than products such as nerve conduit and blood vessel, although sub-clusters related to the suppression of immune rejection by mesenchymal stem cells and cardiac tissue creation using iPS cells exist. These results suggested that research topics obtained from the citation network analysis of interdisciplinary fields are individual basic technologies forming future medical products, and not the medical products themselves.

Citation network analysis, which enables objective evaluation of a large data of research articles, is useful as a primary screening for horizon scanning to investigate research and development trends in medical fields. This study suggested that identifying the technologies that constitute the target research field and carefully checking the trends of research progress is necessary for the analysis of interdisciplinary fields. Furthermore, the integration process of technologies is crucial because the development proceeds by combining technologies in several different areas of expertise. Interpretation of the analysis results will need input from experts in the targeted fields from time to time.

As noted in our previous studies [12, 13], citation network analysis is useful as a primary screening method for horizon scanning because the method is automated and can be easily performed even by people who do not have a high level knowledge or skill in the targeted field. This method can be used for periodical analysis for monitoring the emergence of notable innovative technologies that may require novel regulatory expertise and development of guidance. This study indicated that understanding the development characteristics of a medical product, including the underlying base technologies, and conducting analysis of interdisciplinary fields is necessary for predicting the emergence of medical products with innovative technologies.

### Limitation

This study is based on the analysis of academic articles obtained from the Web of Science, but there are challenges regarding robustness in databases. We considered that the use of Web of Science has a considerable validity from the perspective of comprehensively extracting articles published during the target period, because it has been the sole tool for citation analysis until the launch of Scopus and Google Scholar in 2004. Web of Science is still one of the most effective databases in the historical field, as it has a longer recording period than Scopus [88]. However, Scopus and PubMed are known as leading databases, and the robustness of the method remains to be evaluated in future studies based on the differences in multiple databases.

The citation network analysis does not always directly reflect the article's content because it depends only on the information of the citation relationship of the article. Although we used text mining, different types of information, including interviews with experts in the subject field, are additionally required for an in-depth study of the content of each article.

Advances in bibliometric analysis and text mining both of which have been evolving rapidly, should be closely monitored. Results can be improved by incorporating state-of-the-art analysis technologies.

## Conclusion

This study revealed that citation network analysis objectively reflects the development history of the rapidly evolving and complex field of tissue engineering. Unlike AI-related and T cell-related fields, the tissue engineering field includes technologies from diverse research fields as components, rendering the determination of each basic technology to be used difficult when it is utilized as a medical product from the clustering results alone. Therefore, additional individual surveys are necessary for each basic technology.

We identified topics related to scaffolds and microfluidics for tissue engineering, which have progressed considerably and are expected to be used as medical products in the future. We could identify research topics that are becoming increasingly prominent using a systematic method and proved that citation network analysis can be used as a primary screening method for the horizon scanning procedure. Furthermore, we concluded that identifying the component technologies of the target area is necessary and the trends in research progress should be considered. In addition, when targeting an interdisciplinary area, the integration process of combining technologies from several research fields is critical for the medical product being developed.

## Supplementary Information

Below is the link to the electronic supplementary material.

Supplementary file1 (DOCX 2369 KB)

## Data Availability

The datasets analyzed during the current study were generated by extracting publication data from Web of Science Core Collection as described in Methods.
