# Supplementary material for: Horizon Scanning in Tissue Engineering Using Citation Network Analysis
Source: Ther Innov Regul Sci. 2023 May 18;57(4):810–22. doi: 10.1007/s43441-023-00529-x (PMC10276778; doi:10.1007/s43441-023-00529-x)
Supplement: Supplementary file 1 — Supplementary file1 (DOCX 2369 KB) [file 43441_2023_529_MOESM1_ESM.docx]

**
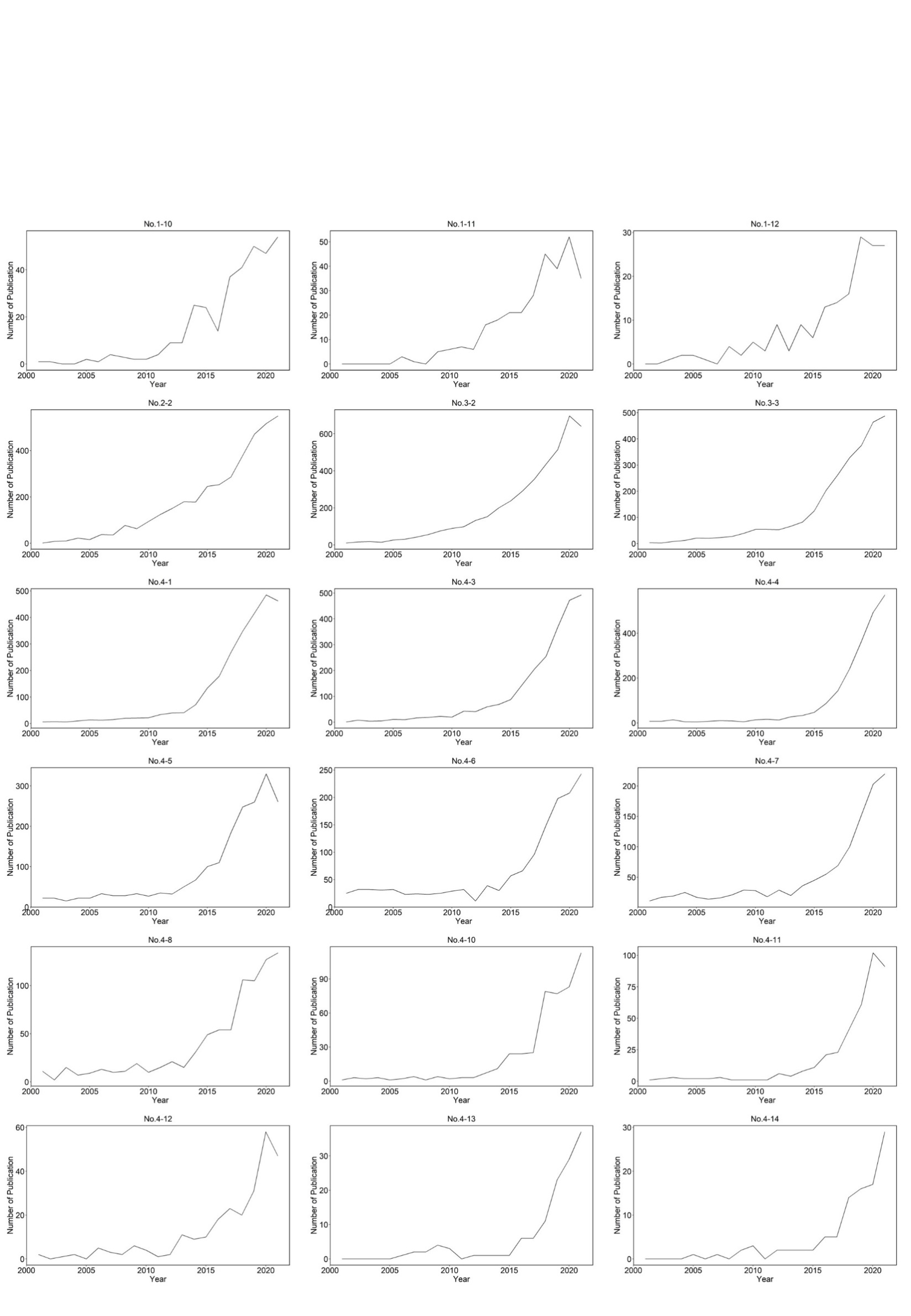
Supplementary material 1-1.**

Among the sub-clusters obtained as a result of the citation network analysis for No. 1–9, those with over 100 articles and 40% of the articles published in 2019–2021 are presented. The horizontal and vertical axes of each graph represent the year of publication of the article and the number of articles published in that year, respectively.


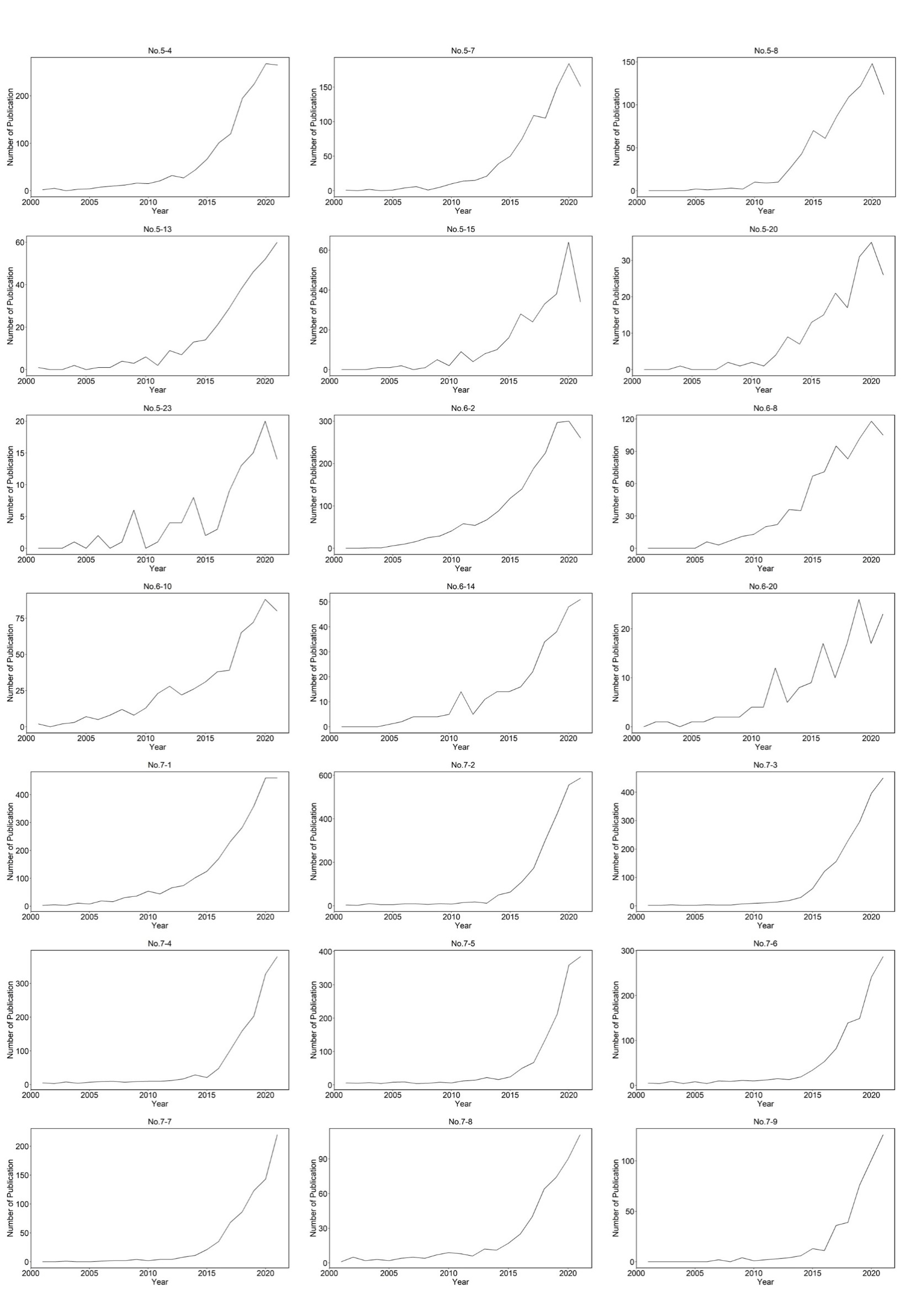


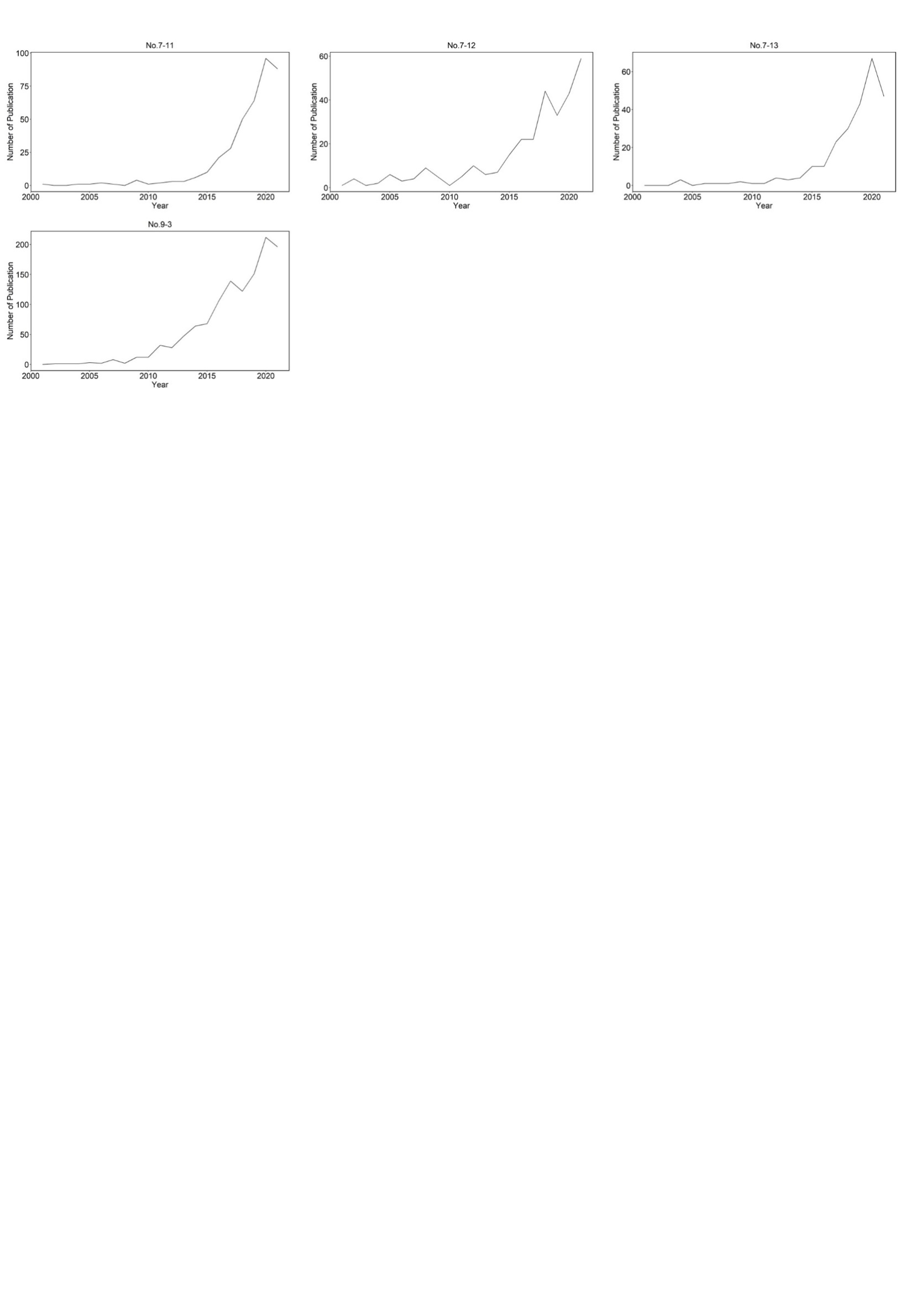


**
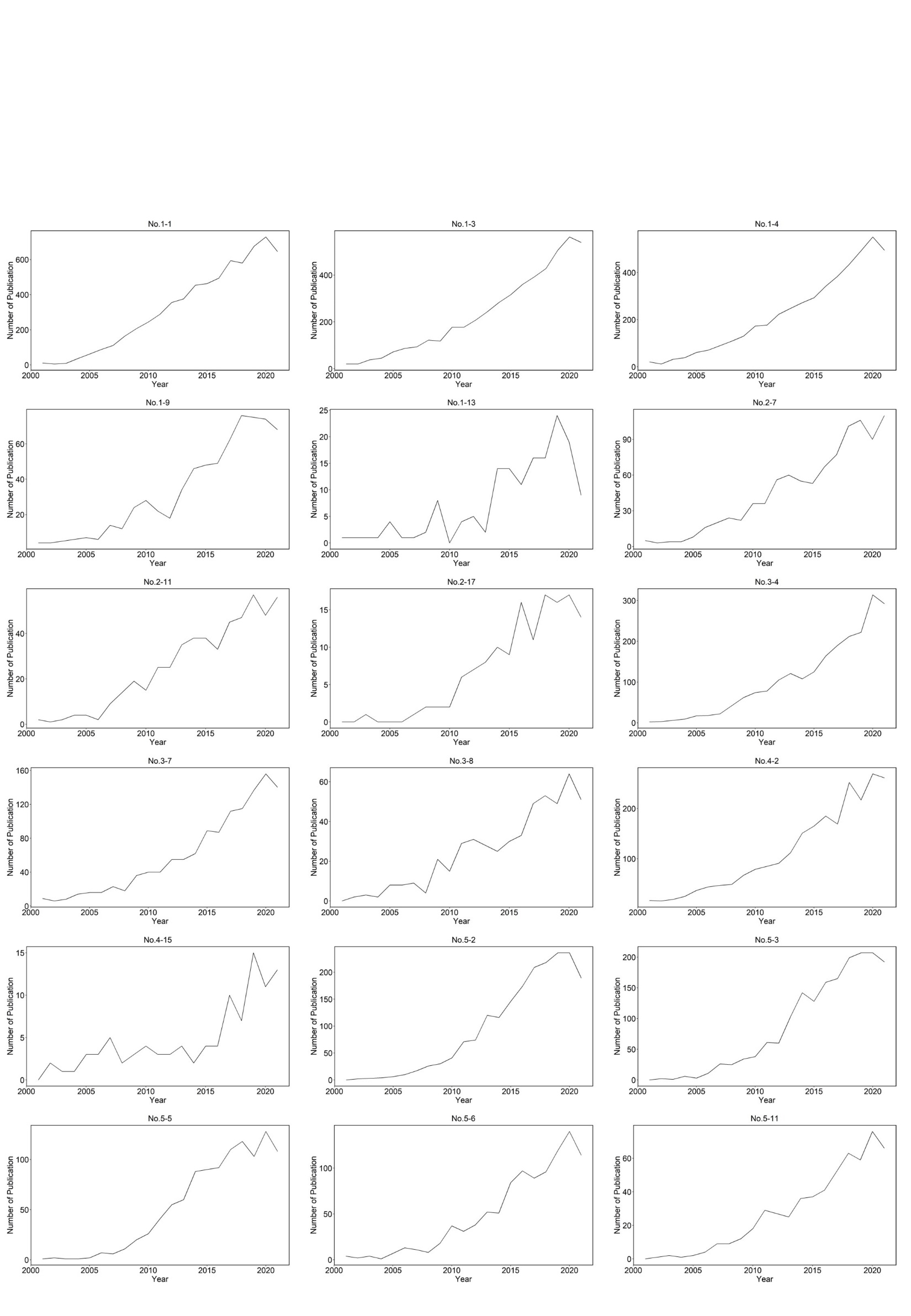
Supplementary material 1-2.**

Among the sub-clusters obtained as a result of the citation network analysis for No. 1–9, those with over 100 articles and 30%–40% of the articles published in 2019–2021 are displayed. The horizontal and vertical axes of each graph represent the year of publication of the article and the number of articles published in that year, respectively.


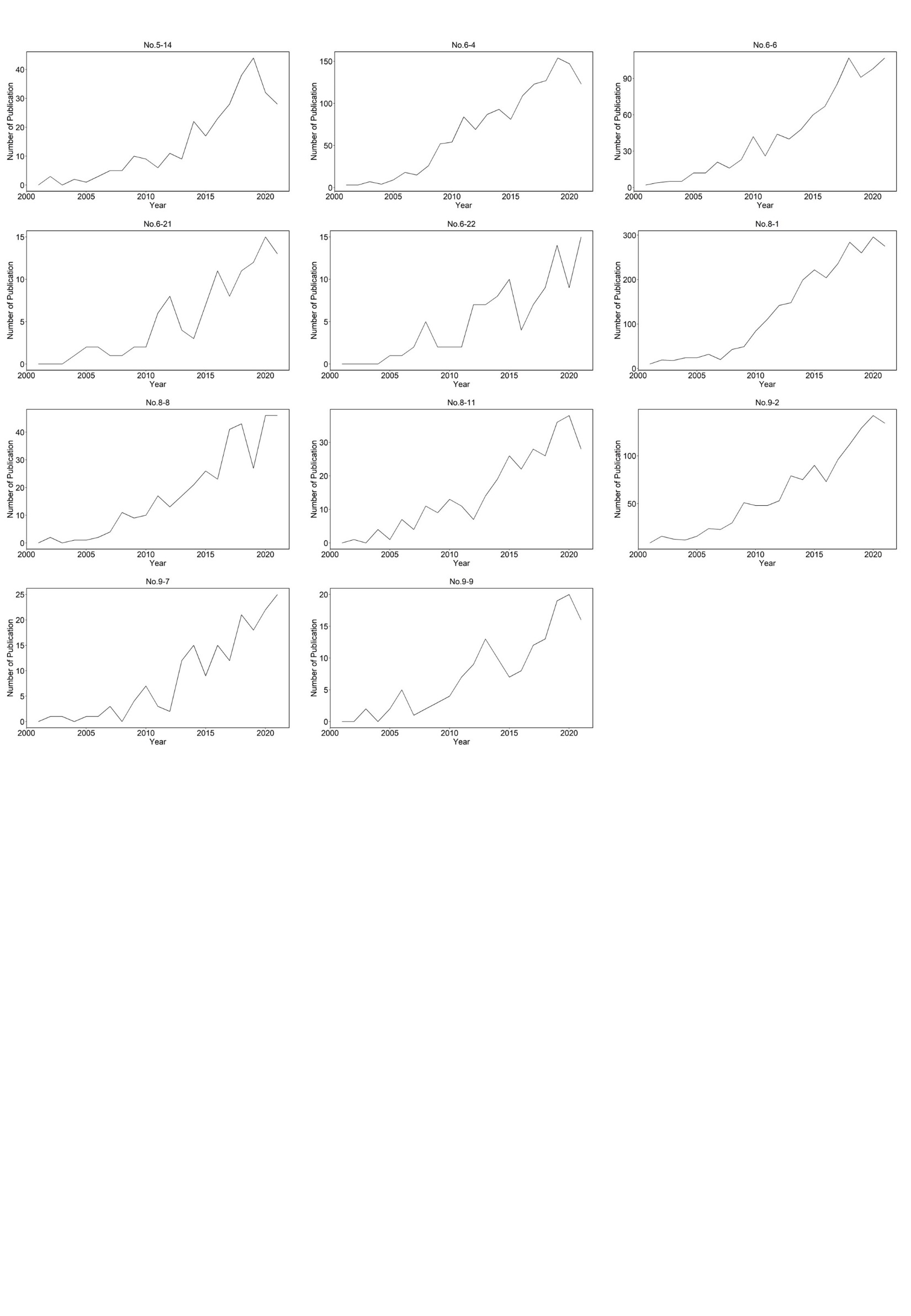


**
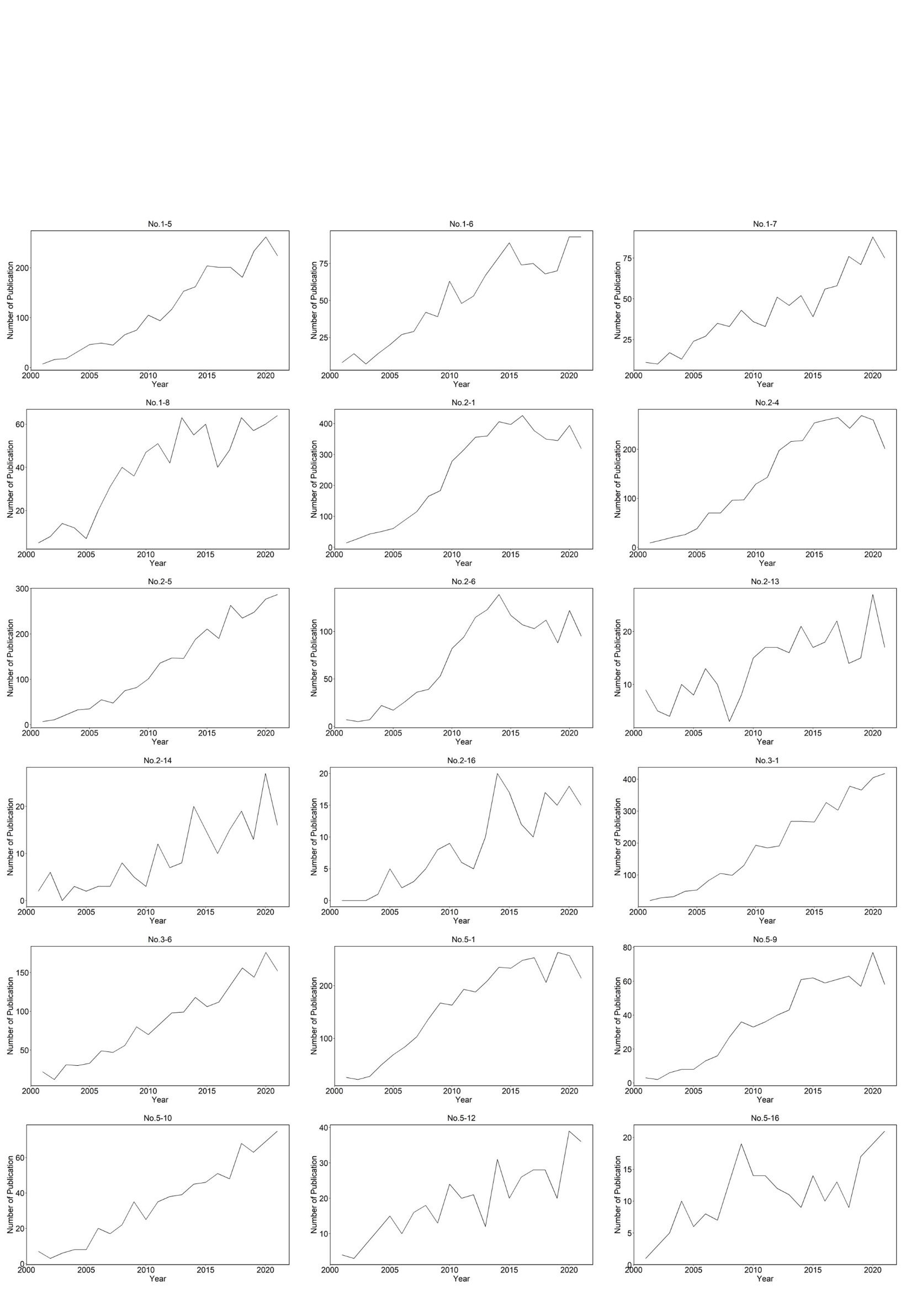
Supplementary material 1-3.**

Among the sub-clusters obtained as a result of the citation network analysis for No. 1–9, those with over 100 articles and 20%–30% of the articles published in 2019–2021 are presented. The horizontal and vertical axes of each graph represent the year of publication of the article and the number of articles published in that year, respectively.


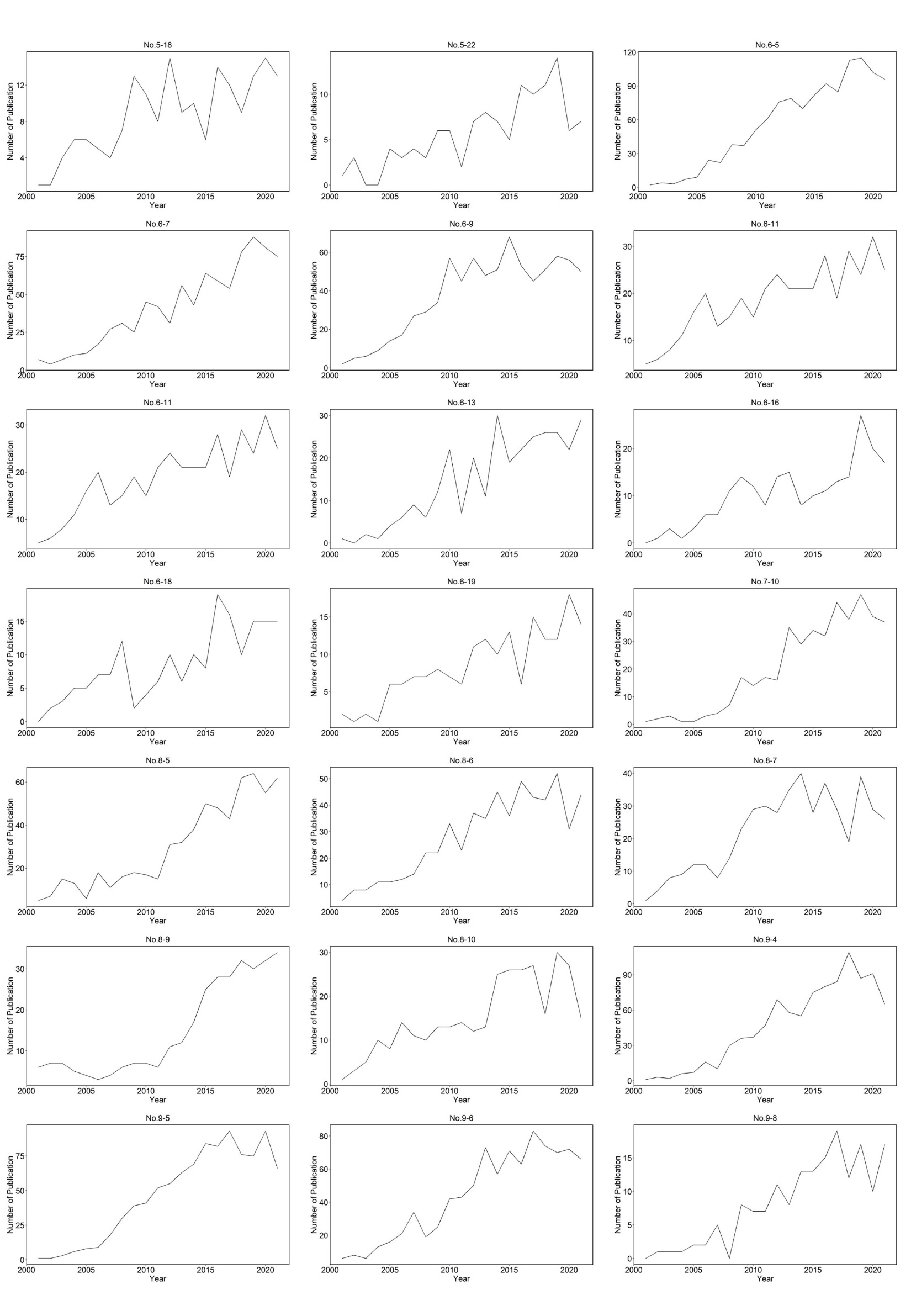


**Supplementary material 2-1.**

Among the results of the citation network analysis of articles published by 2021, sub-clustering was performed on the clusters containing the key articles tracked. The results of the sub-clustering revealed that Articles A-D, I, and J were in sub-cluster 3-3 and Article N was in sub-cluster 3-6.

| **Cluster no.** | **Average year** | **Number of publication** | **Keyword** | **Title of the hub-paper** |
| --- | --- | --- | --- | --- |
| **Cluster 3-1** | 2014.8 | 4245 | Cell, culture, tissue, endothelial, hydrogel, tissue engineering, scaffold, spheroid, microfluidic, endothelial cell | Rapid casting of patterned vascular networks for perfusable engineered three-dimensional tissues |
| **Cluster 3-2** | 2016.8 | 4214 | Hydrogel, tissue engineering, tissue, scaffold, delivery, injectable, polymer, gel, gelatin, drug delivery | Highly stretchable and tough hydrogels |
| **Cluster 3-3** | 2017.3 | 2776 | Bioprinting, hydrogel, printing, scaffold, tissue, construct, bioink, cell, tissue engineering, bioinks | 3D bioprinting of tissues and organs |
| **Cluster 3-4** | 2016.1 | 2216 | Cell, hydrogel, stem cell, stem, tissue, differentiation, tissue engineering, scaffold, matrix, adhesion | The control of human mesenchymal cell differentiation using nanoscale symmetry and disorder |
| **Cluster 3-5** | 2013.3 | 2055 | Hydrogel, scaffold, tissue engineering, cell, tissue, peg, delivery, glycol, matrix, peptide | Synthetic biomaterials as instructive extracellular microenvironments for morphogenesis in tissue engineering |
| **Cluster 3-6** | 2014.0 | 1859 | Nerve, regeneration, scaffold, injury, hydrogel, peripheral nerve, cell, spinal, spinal cord, cord | Neural tissue engineering: Strategies for repair and regeneration |
| **Cluster 3-7** | 2015.4 | 1274 | Alginate, hydrogel, cell, tissue engineering, scaffold, tissue, microfluidic, delivery, microfibers, encapsulation | Alginate: Properties and biomedical applications |
| **Cluster 3-8** | 2015.5 | 519 | Photon, laser, photon polymerization, scaffold, polymerization, hydrogel, fabrication, tissue engineering, microstructures, femtosecond | Ultrafast laser nanostructuring of photopolymers: A decade of advances |

**Supplementary material2-2.**

Among the results of the citation network analysis of articles published by 2021, sub-clustering was performed on the clusters containing the key articles tracked. The results of the sub-clustering revealed that Article G was in sub-cluster 8-9.

| **Cluster no.** | **Average year** | **Number of publication** | **Keyword** | **Title of the hub-paper** |
| --- | --- | --- | --- | --- |
| **Cluster 8-1** | 2015.5 | 2746 | Scaffold, decellularization, ECM, decellularized, tissue, cell, liver, extracellular matrix, matrix, stem cell | An overview of tissue and whole organ decellularization processes |
| **Cluster 8-2** | 2012.5 | 2709 | Vascular, graft, scaffold, vascular graft, endothelial, tissue, cell, tissue engineering, vessel, endothelial cell | Functional arteries grown in vitro |
| **Cluster 8-3** | 2011.5 | 1331 | Valve, heart valve, heart, tissue, scaffold, aortic, leaflet, aortic valve, tissue engineering, tissue engineered | Functional living trileaflet heart valves grown in vitro |
| **Cluster 8-4** | 2012.5 | 1150 | Bladder, tissue, tissue engineering, scaffold, urinary, cell, urethral, muscle, stem cell, stem | Tissue-engineered autologous bladders for patients needing cystoplasty |
| **Cluster 8-5** | 2014.4 | 649 | Intestinal, scaffold, organoids, tissue, stem cell, cell, esophageal, stem, intestine, tissue engineering | Tissue-engineered small intestine improves recovery after massive small bowel resection |
| **Cluster 8-6** | 2013.7 | 601 | Tracheal, trachea, scaffold, tissue, cartilage, tissue engineering, airway, tissue engineered, graft, cell | Clinical transplantation of a tissue-engineered airway |
| **Cluster 8-7** | 2013.5 | 465 | Vocal fold, elastin, vocal, fold, tissue, tissue engineering, hydrogel, scaffold, collagen, ELP | Tissue engineering-based therapeutic strategies for vocal fold repair and regeneration |
| **Cluster 8-8** | 2015.8 | 376 | Macrophage, scaffold, immune, tissue, inflammatory, biomaterials, cell, biomaterial, tissue engineering, implant | Foreign body reaction to biomaterials |

**Supplementary material2-3.**

Among the results of the citation network analysis of articles published by 2021, sub-clustering was performed on the clusters containing key articles tracked. The results of the sub-clustering revealed that Articles L was in sub-cluster 10-4.

| **Cluster No.** | **Average Year** | **Number of Publication** | **Keyword** | **Title of the Hub-paper** |
| --- | --- | --- | --- | --- |
| **Cluster 10-1** | 2014.3 | 1509 | cartilage, chondrogenic, stem cell, stem, mesenchymal, msc, chondrogenesis, articular, cell, differentiation engineering, collagen, bone | Chondrogenic differentiation of adipose-derived adult stem cells in agarose, alginate, and gelatin scaffolds |
| **Cluster 10-2** | 2009.8 | 1221 | cartilage, chondrocytes, articular, tissue, articular cartilage, collagen, construct, scaffold, culture, tissue engineering engineered cartilage, bioreactor | Functional tissue engineering of articular cartilage through dynamic loading of chondrocyte-seeded agarose gels |
| **Cluster 10-3** | 2011.2 | 1008 | cartilage, chondrocytes, tissue, auricular, scaffold, articular, tissue engineering, culture, collagen, chondrocyte cartilage | Transplantation of chondrocytes utilizing a polymer-cell construct to produce tissue-engineered cartilage in the shape of a human ear |
| **Cluster 10-4** | 2014.7 | 887 | cartilage, scaffold, osteochondral, articular, defect, bone, articular cartilage, tissue, repair, tissue engineering mesenchymal stem | Articular cartilage repair: basic science and clinical progress. A review of the current status and prospects |
| **Cluster 10-5** | 2014.5 | 824 | disc, intervertebral, intervertebral disc, ivd, pulposus, nucleus pulposus, degeneration, fibrosus, nucleus, cell | Regenerative effects of transplanting mesenchymal stem cells embedded in atelocollagen to the degenerated intervertebral disc |
| **Cluster 10-6** | 2011.4 | 771 | cartilage, scaffold, chondrocytes, articular, collagen, tissue engineering, chondrocyte, articular cartilage, tissue, hydrogel | Engineering cartilage tissue |
| **Cluster 10-7** | 2013.7 | 481 | meniscus, meniscal, scaffold, tissue, cartilage, knee, tissue engineering, collagen, meniscectomy, repair | The knee meniscus: Structure-function, pathophysiology, current repair techniques, and prospects for regeneration |
| **Cluster 10-8** | 2012.3 | 284 | tmj, cartilage, temporomandibular, temporomandibular joint, disc, tmj disc, joint, tissue engineering, tissue, articular | A self-assembling process in articular cartilage tissue engineering |
